# Animal models and lysosomal pathogenesis of atherosclerosis

**DOI:** 10.48130/animadv-0025-0003

**Published:** 2025-02-25

**Authors:** Zhengchao Wang, Xiang Li, Alexandra K. Moura, Jenny Z. Hu, Yunting Wang, Yang Zhang

**Affiliations:** Department of Pharmacological and Pharmaceutical Sciences, College of Pharmacy, University of Houston, Houston 77204, USA

## Abstract

Atherosclerosis represents a leading cause of cardiovascular disease. Its onset and progression involve complex mechanisms, including inflammation, dysregulation of lipid metabolism, lysosomal dysfunction, and other contributing factors. As intracellular organelles responsible for degradation, lysosomes not only participate in lipid metabolism but also play a pivotal role in foam cell formation and the stabilization of arterial plaques. In recent years, various animal models have been employed to investigate the relationship between lysosomal dysfunction and atherosclerosis, offering novel insights into its pathogenesis and potential therapeutic strategies. This review systematically examines the types of atherosclerosis animal models, the lysosomal mechanisms underlying atherosclerosis, and the prospects and challenges associated with these models. These insights aim to facilitate a deeper understanding of the molecular pathological mechanisms of atherosclerosis and offer novel directions and a robust theoretical foundation for the prevention and treatment of cardiovascular diseases.

## Introduction

Atherosclerosis is characterized by the gradual formation of atherosclerotic plaques on arterial walls. These plaques comprise fat, cholesterol, cellular debris, and calcium^[1,2]^. This pathological process results in arterial stenosis, thereby elevating the risk of cardiovascular conditions such as heart disease and stroke^[3]^. Cardiovascular disease is the leading cause of mortality worldwide, with atherosclerosis serving as a major contributing factor. Approximately 50% of deaths attributed to circulatory system diseases are linked to atherosclerosis, a condition that has become increasingly prevalent in modern society^[4]^.

Atherosclerosis is a global health problem with epidemiological characteristics varying significantly across regions and populations^[5]^. Global epidemiological studies indicate that its incidence and mortality rates are increasing, with cardiovascular deaths constituting a substantial proportion of the total mortality^[5]^. The clinical manifestations of atherosclerosis include ischemic heart disease (IHD), ischemic stroke, and peripheral arterial disease (PAD), which are primary contributors to the global cardiovascular disease burden^[1,6,7]^. The pathological mechanism of atherosclerosis is complex and is closely associated with environmental and genetic risk factors, including hypercholesterolemia, hypertension, smoking, diabetes, and obesity. These factors induce damage and repair responses in the arterial wall, leading to lesion formation^[8,9]^.

Lysosomes, as critical organelles for cellular degradation and metabolism, are crucial to the onset and progression of atherosclerosis through their regulation of autophagy, inflammatory signaling, and lipid metabolism^[10–12]^. Research indicates that lysosomal dysfunction is closely associated with atherosclerosis, resulting in a reduced capacity to process lipids and inflammatory factors^[13–16]^. Moreover, lysosomal activity influences the stability of atherosclerotic plaques, with stable plaques typically linked to intact lysosomal function^[17–19]^.

Lysosomes are membrane-bound organelles within cells, characterized by spherical or vesicular structures with diameters typically ranging from 0.1 to 1 micrometer. Their appearance varies depending on the cell type and functional requirements^[20]^. Lysosomes are enclosed by a phospholipid bilayer membrane, resembling other cellular membranes but containing distinct protein and lipid components^[21]^. The internal cavity of lysosomes is filled with hydrolytic enzymes and degradative molecules, and its pH is typically acidic (approximately pH 4.5 to 5.0). This acidic environment is essential for the optimal activity of hydrolytic enzymes, enabling efficient substrate degradation^[22]^. Lysosomes perform diverse physiological roles, including the degradation and recycling of cellular components, participation in immune responses, regulation of metabolism, signal transduction, and modulation of aging processes^[11,22,23]^. A comprehensive understanding of lysosomal functions is critical for studying atherosclerosis and other diseases, as lysosomal dysfunction is closely associated with the progression of various pathological conditions.

Atherosclerosis is the primary pathological foundation of cardiovascular disease. The study of its pathogenesis and intervention strategies relies heavily on the development and use of animal models^[1,6,7]^. In atherosclerosis animal models, lysosomes play a key role, particularly in modulating inflammatory responses. Lysosomes serve as cellular degradation centers and play a pivotal role in regulating immune responses and inflammatory processes^[10–12]^. Recent technological advancements have led to the development and optimization of various atherosclerosis animal models, offering valuable tools for advancing atherosclerosis research^[24–27]^. Commonly utilized animal models include mice, rabbits, and pigs. These models effectively mimic the progression of human atherosclerosis, serving as critical experimental platforms for research.

## Animal model of atherosclerosis

In atherosclerosis research, various animal models are widely employed to elucidate the disease’s mechanisms and its effects on human health. Atherosclerosis animal models are classified based on animal type, induction methods, severity, and clinical relevance (Table 1). Each model has distinct advantages and limitations, making them suitable for specific research objectives (Table 2)^[24–31]^. This section focuses on commonly used animal models, including hereditary, induced, compound, and other types of atherosclerosis models.

### Hereditary atherosclerosis model

#### ApoE knockout mice

ApoE-deficient mice are among the most widely used models for studying atherosclerosis. Kotsovilis et al. demonstrated that the absence of ApoE results in dysregulated cholesterol metabolism, thereby exacerbating the onset and progression of atherosclerosis^[32]^. Similarly, Getz & Reardon revealed that ApoE-deficient mice exhibited pronounced atherosclerotic features, particularly in the accumulation and impaired clearance of plasma lipoproteins^[33]^. Furthermore, Mulder et al. performed a comparative behavioral analysis of ApoE-deficient and C57Bl/6 mice, showing that ApoE^−/−^ mice exhibited reduced spatial learning and memory efficiency, potentially linked to their heightened susceptibility to neurodegenerative diseases. This study highlights that ApoE is crucial for lipid metabolism and also influences cognitive function^[34]^. Due to the similarity in plaque distribution to humans, these mice are extensively utilized to investigate the molecular mechanisms of atherosclerosis and evaluate therapeutic interventions.

#### LDLR knockout mice

Low-density lipoprotein (LDL) receptor (LDLR)-deficient mice are valuable models for studying atherosclerosis. The lack of the LDL receptor in these mice results in elevated levels of LDL in the bloodstream, which further promotes the development of atherosclerosis. Ngai et al. found that LDLR knockout mice fed a standard rodent diet exhibited significantly decreased food intake and energy expenditure, suggesting that LDLR plays a critical role in regulating energy balance^[35]^. Henninger et al. demonstrated that LDLR knockout mice developed chronic, severe hypercholesterolemia when fed a high-cholesterol diet, with a marked increase in LDL, providing a crucial experimental basis for atherosclerosis research^[36]^. Bieghs et al. discussed how LDLR deletion affects the inflammatory response of the liver, revealing its potential role in atherosclerosis and related diseases^[37]^. Recently, Chaix et al. found that time-restricted feeding combined with a low-cholesterol diet inhibited the progression of atherosclerosis in LDLR knockout mice, offering new insights into dietary interventions, particularly in the prevention and management of cardiovascular diseases^[38]^. This model is particularly suitable for studying cholesterol accumulation and the related pathological mechanisms.

#### ApoE3-Leiden mice

ApoE3-Leiden transgenic mice are generated by introducing the human ApoE3-Leiden gene into C57BL/6 mice. The expression of the human ApoE3 variant, which has a low affinity for LDLR, results in atherosclerosis. Paalvast et al. found that the ApoE3-Leiden transgenic mice markedly impair the clearance of triglyceride-rich lipoproteins, offering valuable insights into lipid metabolism disorders^[39]^. Emini Veseli et al. observed that ApoE3-Leiden mice develop atherosclerosis in the aorta and large blood vessels when fed a Western diet, which is significant for understanding the pathogenesis of human cardiovascular disease^[26]^. Groot et al. demonstrated that ApoE3-Leiden mice exhibited hyperlipidemia and developed pronounced atherosclerosis when fed a high-fat diet^[40]^. Lutgens et al. found that the duration of a high-fat cholesterol diet correlates with the gradual progression of atherosclerosis, highlighting the critical role of diet in arterial health^[41]^. A distinctive feature of these mice is their ability to develop atherosclerosis when fed a high-cholesterol diet, making them valuable for studying various aspects of lipid metabolism.

In addition, the hybrid model of ApoE^−/−^ and diabetic mice (ApoE^−/−/db/db^) showed that lysosomal metabolic dysfunction was especially pronounced in atherosclerosis associated with metabolic syndrome, highlighting the interaction between metabolic disturbances and lysosomal dysfunction^[42]^. Oppi et al.^[43]^ and Galkina et al.^[44]^ found that ApoE^−/−/db/db^ mice at 20 weeks of age exhibited accelerated atherosclerosis progression, along with obesity, insulin resistance, and dysregulated lipid metabolism. Kawashima et al. demonstrated that the combination of ApoE^−/−^ mice and db/db mice significantly enhanced blood glucose control, glucose tolerance, and insulin sensitivity, which was attributed to the absence of ApoE, highlighting its critical role in diabetes pathology^[45]^. Zhang et al.^[46]^ and Hinder et al.^[47]^ found that the combination of ApoE^−/−^ mice and db/db mice exacerbate lipid metabolism disorders. These mice exhibit significant lipid abnormalities, particularly a marked increase in triglyceride levels, simulating the lipid profile of human patients. Additionally, LDLR/ApoA-I double knockout mice exhibited a marked susceptibility to atherosclerosis^[48]^, and Akt2/LDLR double-knockout mice displayed impaired glucose tolerance and reduced atherosclerotic plaque stability^[49]^.

### Induced atherosclerosis model

#### Diet induction model

The diet induction model simulates the development of atherosclerosis by altering the animals’ diet. These models commonly involve high-fat or high-cholesterol diets. For instance, feeding mice a high-fat diet can induce lipid metabolism disorders, leading to pathological changes associated with atherosclerosis. A key advantage of this model is its simplicity and the ability to rapidly observe pathological changes^[50,51]^.

#### Drug induction model

The drug induction model induces atherosclerosis through drug treatments to evaluate their impact on disease progression, using substances such as oleic acid, cholesterol, and others. Research using these models typically investigates the impact of drugs on atherosclerosis progression, focusing on mechanisms such as the regulation of cholesterol metabolism, inflammatory responses, and angiogenesis. These studies offer valuable theoretical insights for the development of novel therapeutic agents^[29,50]^.

Among these, high-fat diet and vitamin D3-induced AS rabbit models have been employed to investigate the role of lysosomes in lipid metabolism and inflammation regulation. Funes et al. found that rabbits exhibit heightened sensitivity to dietary cholesterol, and a high-fat diet promotes liver fat deposition, contributing to atherosclerosis development^[52]^. Li et al. demonstrated that inducing obesity in rabbits via a high-fat diet led to significant increases in blood glucose (GLU), total cholesterol (TC), triglycerides (TG), and LDL cholesterol concentrations, which serve as important biomarkers and provide a pathological basis for atherosclerosis research^[53]^. Mäkitaipalee et al. found that vitamin D3 induces arterial wall thickening, plaque formation, and endothelial dysfunction in rabbits, all of which are typical characteristics of atherosclerosis^[54]^.

### Compound atherosclerosis model

#### Arterial ligation model

The arterial ligation model is widely used to study various physiological and pathological conditions, including cardiovascular diseases, reproductive system disorders, and drug development. Local ligation or injury of arteries to simulate vascular stenosis is useful for studying the mechanisms of lesion formation; however, the morphology and stability of the resulting plaques are typically suboptimal. In 2004, Myers & Liaw conducted an extensive analysis of the vascular remodeling response following carotid artery ligation using a multivariate approach. The study emphasized the role of arterial ligation in inducing vascular remodeling, which is crucial for understanding arteriosclerosis and other vascular pathologies. This study offers new insights into the application of arterial ligation models in vascular biology^[55]^.

#### Arteriovenous fistula model

The arteriovenous fistula (AVF) model creates abnormal channels between arteries and veins to simulate the exacerbating effect of disrupted blood flow on atherosclerosis. The AVF model established by Croatt et al. observed alterations in the vascular endothelium and their impact on hemodynamics^[56]^. Bozzetto et al. demonstrated that the autologous AVF is the preferred vascular access for hemodialysis^[57]^. Li et al. explored the foundational research and clinical significance of AVF models, highlighting the use of experimental mouse AVF models in studying vascular biology and complications related to hemodialysis^[58]^.

### Other atherosclerosis models

In addition to mice, other animal species have also been employed in atherosclerosis research. For example, rabbits are particularly sensitive to high-cholesterol diets and are frequently used in studies evaluating the efficacy of drugs in treating atherosclerosis. The physiology and anatomical structure of porcine blood vessels closely resemble those of humans, making pigs a common model in cardiovascular disease studies.

#### Rat model

Rats are also used in atherosclerosis research, particularly in models induced by high-cholesterol diets. For example, Zhao et al.^[59]^, Wu et al.^[60]^, and Lee et al.^[61]^ demonstrated that the progression of human atherosclerosis can be simulated in rats through dietary and lifestyle modifications, thus aiding in the understanding of the disease mechanism. In addition, Lee et al. developed ApoE knockout rats to investigate the initiation mechanisms of human atherosclerosis^[61]^.

#### Rabbit model

The rabbit is one of the earliest animal models used in atherosclerosis research^[62]^. Rabbits can rapidly form arterial plaques after being fed high-cholesterol or Western diets, making them widely employed in basic research and drug development^[63,64]^. A key advantage of this model is that its physiological characteristics closely resemble those of humans, particularly in cholesterol metabolism^[65]^.

#### Pig model

The cardiovascular system of pigs closely resembles that of humans. Pigs are commonly used to evaluate the efficacy of drugs in the treatment of atherosclerosis, making them an ideal model for studying human atherosclerosis^[66]^. Kim et al. and Hoogendoorn et al. showed that coronary atherosclerotic lesions in pigs are similar to those in humans, providing a valuable model for studying the role of inflammation in atherosclerosis^[67,68]^.

#### Non-human primate model

In the study of atherosclerosis, non-human primates, such as macaques and rhesus monkeys, are regarded as the most clinically relevant model due to their similar physiological structure and pathological characteristics to humans^[69]^. Cox et al. showed that these animals can develop atherosclerotic plaques similar to those in humans under diet induction, which is highly valuable for studying the clinical manifestations and therapeutic effects of the disease^[70]^.

#### Fish model

Zebrafish have emerged as a promising atherosclerosis model, gaining significant attention in recent years. Compared to mice, zebrafish have a smaller body size, and their embryonic and juvenile stages are transparent, allowing for the direct observation of physiological changes. This model has demonstrated potential in drug screening and gene function studies^[71,72]^.

## Lysosomal mechanism of atherosclerosis

Patients with atherosclerosis frequently exhibit lysosomal dysfunction, leading to the excessive accumulation of intracellular lipids, which in turn promotes the progression of the disease^[13]^. Bhat et al.^[14]^ and Zhang et al.^[17]^ suggested that atherosclerosis can be considered, to some extent, an acquired lysosomal storage disorder.

### The role of lysosomes in the formation of foam cells

Foam cells serve as key markers of early atherosclerosis, with their formation reliant on the uptake and degradation of LDL by macrophages. LDL cholesterol penetrates the vessel wall via endothelial cell disruptions, where macrophages internalize it through phagocytosis. These LDL particles are then transported into lysosomes for degradation. Under normal conditions, lysosomes effectively degrade these lipids, but in atherosclerosis, macrophage lysosomal function is impaired, resulting in lipid metabolism dysregulation^[13,15,73]^.

Macrophages exhibit a dual role at various stages of atherosclerosis. During the early stages of atherosclerosis, they protect blood vessels by engulfing LDL and clearing cellular debris. However, during disease progression, lysosomal function in macrophages is compromised, driving their transformation into foam cells. These foam cells not only damage surrounding endothelial cells but also promote the migration and proliferation of smooth muscle cells, leading to fiber cap formation and further exacerbating plaque instability^[74]^.

Disruption of the lysosomal acidic environment impairs the degradation of cholesterol esters, promoting the accumulation of undigested lipids within cells. The disruption of the acidic environment within atherosclerotic plaques may hinder lipid and protein degradation, accelerating foam cell formation. For example, an increase in lysosomal pH is considered a key factor in foam cell formation^[75]^.

### Lysosomes and inflammation regulation

Atherosclerosis is an inflammation-driven disease. Upon stimulation by oxidized LDL, endothelial cells release pro-inflammatory cytokines, which recruit monocytes and lymphocytes to the site of injury. Once monocytes differentiate into macrophages, they further engulf lipids and release inflammatory mediators, processes closely linked to changes in lysosomal function^[76]^.

Inflammation is crucial in the onset and progression of atherosclerosis, primarily involving macrophage infiltration, cytokine secretion, and endothelial inflammation^[76,77]^. A large accumulation of macrophages in the arterial wall not only engulfs lipids but also secretes various cytokines, further promoting local inflammatory responses. Macrophages and other immune cells release inflammatory mediators, such as tumor necrosis factor and interleukins, which exacerbate endothelial damage and plaque formation^[78]^. In the inflammatory state, endothelial cells express increased adhesion molecules, promoting leukocyte adhesion and infiltration, which further advances the progression of atherosclerosis^[79]^.

Lysosomes are also crucial in the inflammation associated with atherosclerosis. They regulate the activity of macrophages, which are key players in the formation and progression of the disease. Under normal conditions, lysosomes enable macrophages to effectively clear cellular debris and apoptotic cells, maintaining local environmental stability. However, lysosomal dysfunction impairs macrophage phagocytosis, exacerbating the inflammatory response and accelerating the progression of atherosclerosis^[18]^.

### Lysosomal autophagy and cell apoptosis

Autophagy is a critical cellular process through which damaged or unnecessary components are cleared by lysosomes. Dysfunction of autophagy is closely associated with the onset and progression of atherosclerosis^[80]^. In ApoE^−/−^ mice, the knockout of autophagy-related genes, such as LC3 and ATG5, significantly accelerates the formation of atherosclerotic plaques. Defects in lysosomal function, including decreased lysosomal enzyme activity, impair autophagy, increase cell apoptosis, and exacerbate damage to the arterial wall^[13]^. Enhancing macrophage autophagy can slow the progression of atherosclerosis, suggesting that the functional restoration of lysosomes may represent a promising therapeutic strategy for treating the disease^[81]^.

## Lysosomal research in atherosclerosis animal models

The role of lysosomal function in atherosclerosis has become a major research focus. Lysosomes are critical in the initiation and progression of atherosclerotic lesions by regulating cellular metabolism, autophagy, and inflammatory responses.

### Lysosomal research in the ApoE^−/−^ mouse model

In the ApoE^−/−^ mouse model, disruptions in the lysosomal acidic environment and reduced enzyme activity in arterial wall macrophages are key factors contributing to foam cell formation^[82,83]^. Furthermore, lysosomes are critical in modulating inflammation in ApoE^−/−^ models, primarily through their role in autophagy, inflammasome regulation, efferocytosis, and lipid metabolism^[13,84,85]^. Hence, therapeutic strategies that enhance lysosomal functions or reduce the production of pro-inflammatory cytokines, provide a potential approach to mitigate atherosclerosis in these models. He et al. demonstrated that neutralizing pro-inflammatory cytokines, such as tumor necrosis factor-alpha (TNF-α), interleukin-1*β* (IL-1*β*), and IL-6, can suppress inflammatory responses, suggesting that targeting these cytokines may offer a new therapeutic approach in ApoE^−/−^ mice^[86]^. Mangarova et al. showed that the absence of IL-1*β* significantly reduced the severity of atherosclerosis, indicating that inhibiting IL-1*β* could improve vascular health in ApoE^−/−^mice^[87]^. Furthermore, Grebe et al. found that IL-1 receptor antagonists (IL-1Ra) inhibited the formation of fat streaks in ApoE^−/−^ mice^[88]^. To better replicate human pathological conditions, researchers have developed atherosclerosis models with metabolic syndrome or diabetes, such as the ApoE^−/−/db/db^ hybrid mouse model, to investigate the interplay between metabolic disorders and atherosclerosis^[42–45]^.

### Lysosomal research in the LDLR^−/−^ mouse model

In the LDLR^−/−^ mouse model, reduced activity of lysosomal lipid metabolic enzymes, such as acid cholesterol esterase, leads to an increase in atherosclerotic plaques and plaque instability^[14,89]^. Oxidative stress further exacerbates these pathological changes by promoting macrophage foam cell formation and lysosomal dysfunction. For instance, Rodger et al. found that LDLR deficiency results in decreased expression of antioxidant enzymes, making these mice more vulnerable to oxidative damage^[90]^. Wu et al. demonstrated that Liraglutide protects against LOX-1-mediated oxidative stress and inflammation in LDLR^−/−^ mice through GLP-1 receptor-dependent downregulation, suggesting that modulating oxidative stress can improve cardiovascular health^[91]^.

### Lysosomal research in other animal models

In a high glucose environment, the stability of lysosomal membranes is compromised, leading to increased macrophage apoptosis and the establishment of a diabetes-atherosclerosis model. For example, Wang et al. found that the incidence of cardiovascular complications in diabetic patients was significantly higher than in non-diabetic individuals, with diabetes-induced atherosclerotic plaques being more prone to rupture, thus increasing the risk of cardiovascular events^[92]^. Additionally, the use of CRISPR/Cas9 technology to target the knockout of pro-inflammatory or metabolic genes, such as PCSK9, in mouse models has been shown to significantly reduce atherosclerosis. Bao et al. demonstrated that PCSK9 overexpression induced atherosclerosis and associated pathological changes, suggesting that PCSK9 plays a critical role in the pathogenesis of atherosclerosis^[93]^. Shin et al. further found that PCSK9 not only impacts arterial health through traditional cholesterol metabolism but also accelerates atherosclerosis by promoting inflammatory responses^[94]^.

Notably, gene editing tools for large animals have made it possible to create genetically modified minipigs that share many similarities with humans in terms of lesion site and histopathological preferences. For example, minipigs with liver-specific expression of human D374Y-PCSK9 exhibit severe hypercholesterolemia and progressive atherosclerotic lesions^[95,96]^. Together with existing porcine atherosclerosis models based on spontaneous mutations or severe diabetes, these models provide new approaches for translational research in atherosclerosis^[97,98]^.

In addition, the use of non-human primates, such as laboratory monkeys, for atherosclerosis modeling is very expensive, highly regulated, and requires very specialized laboratory animal science skills. Therefore, these models are not often used.

## Prospects and challenges of atherosclerosis animal models

The formation mechanisms, metabolic environments, and immune responses in atherosclerosis animal models do not perfectly replicate those of humans. Therefore, future research should focus on developing primate models that more closely resemble human pathological characteristics. Currently, most animal models rely on rodents such as mice and rats, which exhibit significant differences in biological traits and genetic backgrounds compared to humans. This discrepancy means that findings from animal models may not always be directly translatable to clinical settings^[24–31]^. However, with advancements in gene editing technologies, such as CRISPR-Cas9, researchers can create more precise gene deletion or mutation models to simulate the specific genetic profiles associated with human atherosclerosis^[93,94]^.

A single animal model cannot fully capture the complexity and diversity of atherosclerosis. Therefore, future research should combine genetic, chemical, and trauma-based models to better support studies on the entire disease progression. While current models primarily focus on small animals like mice and rats, these models often fail to replicate the full range of pathological and physiological characteristics seen in humans. As such, incorporating larger animal models, such as pigs and rabbits, which more closely resemble human lipid metabolism and vascular lesion morphology^[64,66]^, will enhance our understanding of atherosclerosis development.

Currently, evaluation criteria for atherosclerosis models predominantly focus on morphological aspects, with functional assessments receiving less attention. In the future, it will be essential to develop evaluation indicators that incorporate functional, metabolic, and molecular signaling factors. Atherosclerosis research should emphasize translational medicine, using insights from animal models to inform clinical practice. For instance, studying early atherosclerosis through rat models can provide valuable information for understanding early disease intervention^[59–61]^. Furthermore, creating animal models that closely mimic human physiological and pathological characteristics will enhance the clinical translation of research findings, fostering the development and application of new therapies.

It is important to highlight that the lysosomal mechanism of atherosclerosis offers both a theoretical foundation and practical guidance for lysosomal function-targeted therapies. Zhang et al.^[17]^ and Sergin et al.^[99]^ demonstrates that lysosomal dysfunction not only plays a role in the onset and progression of atherosclerosis but also presents a novel therapeutic target for the disease. As a result, lysosomal enzyme activators and stabilizers of the lysosomal acidic environment are considered to have promising therapeutic potential.

## Summary and prospects

Animal models are indispensable in atherosclerosis research, enabling the exploration of specific research questions and providing valuable insights into the molecular mechanisms of the disease. Through the study of animal models such as mice, rabbits, and pigs, we can deepen our understanding of atherosclerosis and develop potential prevention strategies. While animal models offer significant insights, caution is needed when translating these findings to human contexts to ensure the reliability and validity of results. Furthermore, the role of lysosomes in atherosclerosis formation is critical; they not only participate in lipid metabolism and processing but also play a central role in regulating the inflammatory response. As research in this area progresses, targeting lysosomal function may offer new therapeutic avenues for the prevention and treatment of atherosclerosis. Ultimately, this research will not only enhance our understanding of atherosclerosis but also serve as a crucial reference for studying other related cardiovascular diseases.

## Figures and Tables

**Table 1.** Classification of atherosclerosis animal models.

Classification criteria	Animal model name	Characteristics of animal models
Classification based on animal species	Mouse model	The most commonly used animal models in atherosclerotic research. A variety of missing genes or transgenic mice created by genetic engineering technology. Different physiological characteristics in atherosclerotic studies, such as LDLR^−/−^ mice.
	Rat model	The commonly used animal models in atherosclerotic research. The unique advantages for surgery and observation.
	Pig model	The structure of porcine coronary artery is similar to that of human, which is of great significance in atherosclerotic research. Pig models can better simulate human physiological and pathological characteristics, especially in drug testing and interventional therapy.
	Other large animal models	The other large animals also used for atherosclerotic research, such as monkeys. These animal models pose ethical and cost challenges.
Classification based on the induction model method	Genetic engineering model	These models induce atherosclerosis through gene manipulation, such as gene knockout or transgene. LDLR^−/−^ and ApoE^−/−^ models are constructed by genetic engineering, which can simulate the pathogenesis of human atherosclerosis.
	Dietary induction model	Atherosclerosis can be induced by changing the diet of animals, especially the diet with high fat and cholesterol. This model is relatively simple and does not require complex genetic manipulation.
	Chemical induction model	Atherosclerosis can be induced by injecting chemicals, such as vitamin D3, carbon tetrachloride, etc. These chemicals can cause endothelial damage and inflammatory reaction, thus promoting the formation of atherosclerosis.
	Mechanical damage model	The atherosclerotic progression can be observed by applying mechanical damage to the blood vessels, such as the damage to the intima of the arteries. This model can be used to study the repair of vascular intima and the follow-up progress of atherosclerosis.
Classification based on the severity of the model	Mild atherosclerosis model	These models generally exhibit early lesions and are commonly used to study the early mechanisms and prevention strategies of diseases. The characteristics of mild lesions are less lipid deposition and less obvious endometrial hyperplasia.
	Moderate atherosclerosis model	In these models, lipid deposition is evident within the arteries, accompanied by a certain degree of intimal hyperplasia. The moderate model is suitable for studying the progression of diseases and the effectiveness of related treatment methods.
	Severe atherosclerosis model	Severe models with obvious plaque formation and vascular lumen stenosis are usually used to study the complications and clinical manifestations of atherosclerosis. This type of model is very important for drug development and efficacy evaluation.
Classification based on clinical relevance	Human disease model	These models are designed to simulate the characteristics of human atherosclerosis, including plaque formation, rupture and the resulting clinical consequences. Through these models, researchers can better understand the mechanisms of human diseases.
	Non-human disease models	These models may not fully reflect the pathological characteristics of humans, but they provide useful information in certain aspects such as physiological responses, drug metabolism, etc. This type of model is equally important in basic research and drug development.

**Table 2.** Comparison of advantages and limitations of atherosclerosis animal models.

Animal model		Advantages and limitations of atherosclerosis animal models
Mouse model	Advantages	Convenience of genetic engineering: specific gene deletion/mutation mice can be created by gene editing to study the role of these genes in atherosclerosis. Fast reproductive speed: mice have a short reproductive cycle and can quickly obtain experimental samples for long-term and large-scale research. Relatively low cost: compared with other large animals, the feeding and management costs of mice are lower, making large-scale experiments more feasible.
	Limitations	Physiological differences with humans: although mice can be manipulated at the genetic level, their physiological structure and metabolic processes differ significantly from humans. Apparent characteristics of atherosclerosis: the symptoms of atherosclerosis may not be as obvious in some mouse models as that of human beings, especially in the intervention of high-fat diet.
Rat model	Advantages	Larger body size: rats have a larger body size compared to mice, making them easier for surgery and organ sampling. More complex physiological characteristics: rats are closer to humans in terms of physiology and biochemistry, and can better simulate human pathological states.
	Limitations	Long reproductive cycle: the reproductive cycle of rats is relatively long, and the speed of obtaining experimental samples is slower. Higher cost: the feeding cost of rats is higher compared to mice, which may limit the conduct of large-scale experiments.
Rabbit model	Advantages	Spontaneous occurrence of atherosclerosis: rabbits are prone to spontaneously develop atherosclerosis under a high-fat diet, which is a good model for observing natural pathological process. Similarity of cardiovascular system: the cardiovascular system of rabbits is similar to that of humans, providing more relevant physiological data.
	Limitations	High feeding costs: the cost of raising and managing rabbits is high, which limits the expansion of experimental scale. Ethical issues of research: as rabbits are larger mammals, the ethical issues involved may be more complex.
Pig model	Advantages	Anatomical similarity with humans: the cardiovascular anatomical structure of pigs is relatively similar to that of humans and is an important model for studying cardiovascular diseases. Spontaneous atherosclerosis: pigs can spontaneously develop atherosclerosis under appropriate diet conditions, providing an opportunity to observe the progress of disease.
	Limitations	The extremely high cost of raising and managing pigs: the cost of raising and managing pigs is very high due to their large size, which limits their application in certain research. The longer reproductive cycle: pigs have a longer reproductive cycle, and the speed of obtaining experimental samples is slower than that of mice and rats.

## Data Availability

Data sharing not applicable to this article as no datasets were generated or analyzed during the current study.

## References

[1] Libby P, Buring JE, Badimon L, Hansson GK, Deanfield J, 2019. Atherosclerosis. Nature Reviews Disease Primers 5:56 10.1038/s41572-019-0106-z 31420554

[2] Lusis AJ. 2000. Atherosclerosis. Nature 407:233–41 10.1038/35025203 PMC2826222 11001066

[3] Jebari-Benslaiman S, Galicia-García U, Larrea-Sebal A, Olaetxea JR, Alloza I, 2022. Pathophysiology of atherosclerosis. International Journal of Molecular Sciences 23:3346 10.3390/ijms23063346 PMC8954705 35328769

[4] Luca AC, David SG, David AG, \Țarcă V, Pădureț IA, 2023. Atherosclerosis from newborn to adult-epidemiology, pathological aspects, and risk factors. Life 13:2056 10.3390/life13102056 PMC10608492 37895437

[5] Herrington W, Lacey B, Sherliker P, Armitage J, Lewington S. 2016. Epidemiology of atherosclerosis and the potential to reduce the global burden of atherothrombotic disease. Circulation Research 118:535–46 10.1161/CIRCRESAHA.115.307611 26892956

[6] Falk E. 2006. Pathogenesis of atherosclerosis. Journal of the American College of Cardiology 47:C7–C12 10.1016/j.jacc.2005.09.068 16631513

[7] Rafieian-Kopaei M, Setorki M, Doudi M, Baradaran A, Nasri H. 2014. Atherosclerosis: process, indicators, risk factors and new hopes. International Journal of Preventive Medicine 5:927–46 PMC4258672 25489440

[8] Insull W. 2009. The pathology of atherosclerosis: plaque development and plaque responses to medical treatment. The American Journal of Medicine 122:S3–S14 10.1016/j.amjmed.2008.10.013 19110086

[9] Gusev E, Sarapultsev A. 2023. Atherosclerosis and inflammation: insights from the theory of general pathological processes. International Journal of Molecular Sciences 24:7910 10.3390/ijms24097910 PMC10178362 37175617

[10] Zhang Z, Yue P, Lu T, Wang Y, Wei Y, 2021. Role of lysosomes in physiological activities, diseases, and therapy. Journal of Hematology & Oncology 14:79 10.1186/s13045-021-01087-1 PMC8120021 33990205

[11] Xu H, Ren D. 2015. Lysosomal physiology. Annual Review of Physiology 77:57–80 10.1146/annurev-physiol-021014-071649 PMC4524569 25668017

[12] Appelqvist H, Wäster P, Kågedal K, Öllinger K. 2013. The lysosome: from waste bag to potential therapeutic target. Journal of Molecular Cell Biology 5:214–26 10.1093/jmcb/mjt022 23918283

[13] Marques ARA, Ramos C, Machado-Oliveira G, Vieira OV. 2021. Lysosome (dys)function in atherosclerosis-a big weight on the shoulders of a small organelle. Frontiers in Cell and Developmental Biology 9:658995 10.3389/fcell.2021.658995 PMC8039146 33855029

[14] Bhat OM, Li PL. 2021. Lysosome function in cardiovascular diseases. Cellular Physiology and Biochemistry 55:277–300 10.33594/000000373 PMC8743031 34019755

[15] Emanuel R, Sergin I, Bhattacharya S, Turner J, Epelman S, 2014. Induction of lysosomal biogenesis in atherosclerotic macrophages can rescue lipid-induced lysosomal dysfunction and downstream sequelae. Arteriosclerosis, Thrombosis, and Vascular Biology 34:1942–52 10.1161/ATVBAHA.114.303342 PMC4140993 25060788

[16] Wojtasińska A, Frąk W, Lisińska W, Sapeda N, Młynarska E, 2023. Novel insights into the molecular mechanisms of atherosclerosis. International Journal of Molecular Sciences 24:13434 10.3390/ijms241713434 PMC10487483 37686238

[17] Zhang X, Misra SK, Moitra P, Zhang X, Jeong SJ, 2023. Use of acidic nanoparticles to rescue macrophage lysosomal dysfunction in atherosclerosis. Autophagy 19:886–903 10.1080/15548627.2022.2108252 PMC9980706 35982578

[18] Skeyni A, Pradignac A, Matz RL, Terrand J, Boucher P. 2024. Cholesterol trafficking, lysosomal function, and atherosclerosis. American Journal of Physiology Cell Physiology 326:C473–86 10.1152/ajpcell.00415.2023 38145298

[19] Popa-Fotea NM, Ferdoschi CE, Micheu MM. 2023. Molecular and cellular mechanisms of inflammation in atherosclerosis. Frontiers in Cardiovascular Medicine 10:1200341 10.3389/fcvm.2023.1200341 PMC10434786 37600028

[20] Trivedi PC, Bartlett JJ, Pulinilkunnil T. 2020. Lysosomal biology and function: modern view of cellular debris bin. Cells 9:1131 10.3390/cells9051131 PMC7290337 32375321

[21] Saftig P, Klumperman J. 2009. Lysosome biogenesis and lysosomal membrane proteins: trafficking meets function. Nature Reviews Molecular Cell Biology 10:623–35 10.1038/nrm2745 19672277

[22] Yang C, Wang X. 2021. Lysosome biogenesis: regulation and functions. The Journal of Cell Biology 220:e202102001 10.1083/jcb.202102001 PMC8105738 33950241

[23] Patra S, Patil S, Klionsky DJ, Bhutia SK. 2023. Lysosome signaling in cell survival and programmed cell death for cellular homeostasis. Journal of Cellular Physiology 238:287–305 10.1002/jcp.30928 36502521

[24] Gisterå A, Ketelhuth DFJ, Malin SG, Hansson GK. 2022. Animal models of atherosclerosis-supportive notes and tricks of the trade. Circulation Research 130:1869–87 10.1161/CIRCRESAHA.122.320263 35679358

[25] Lee YT, Laxton V, Lin HY, Chan YWF, Fitzgerald-Smith S, 2017. Animal models of atherosclerosis. Biomedical Reports 6:259–66 10.3892/br.2017.843 PMC5403338 28451383

[26] Emini Veseli B, Perrotta P, De Meyer GRA, Roth L, Van der Donckt C, 2017. Animal models of atherosclerosis. European Journal of Pharmacology 816:3–13 10.1016/j.ejphar.2017.05.010 28483459

[27] Fuster JJ, Castillo AI, Zaragoza C, Ibáñez B, Andrés V. 2012. Animal models of atherosclerosis. Progress in Molecular Biology and Translational Science 105:1–23 10.1016/B978-0-12-394596-9.00001-9 22137427

[28] van der Vaart JI, van Eenige R, Rensen PCN, Kooijman S. 2024. Atherosclerosis: an overview of mouse models and a detailed methodology to quantify lesions in the aortic root. Vascular Biology 6:e230017 10.1530/VB-23-0017 PMC11046329 38428154

[29] Daugherty A, Tall AR, Daemen MJAP, Falk E, Fisher EA, 2017. Recommendation on design, execution, and reporting of animal atherosclerosis studies: a scientific statement from the American heart association. Arteriosclerosis, Thrombosis, and Vascular Biology 37(9):e131–e157 10.1161/ATV.0000000000000062 28729366

[30] Zhang Y, Fatima M, Hou S, Bai L, Zhao S, 2021. Research methods for animal models of atherosclerosis (Review). Molecular Medicine Reports 24:871 10.3892/mmr.2021.12511 PMC8569513 34713295

[31] Lee YT, Lin HY, Chan YWF, Li KHC, To OTL, 2017. Mouse models of atherosclerosis: a historical perspective and recent advances. Lipids in Health and Disease 16:12 10.1186/s12944-016-0402-5 PMC5240327 28095860

[32] Kotsovilis S, Salagianni M, Varela A, Davos CH, Galani IE, 2024. Comprehensive analysis of 1-year-old female apolipoprotein E-deficient mice reveals advanced atherosclerosis with vulnerable plaque characteristics. International Journal of Molecular Sciences 25:1355 10.3390/ijms25021355 PMC10816800 38279355

[33] Getz GS, Reardon CA. 2016. ApoE knockout and knockin mice: the history of their contribution to the understanding of atherogenesis. Journal of Lipid Research 57:758–66 10.1194/jlr.R067249 PMC4847636 27015743

[34] Mulder M, Blokland A, van den Berg DJ, Schulten H, Bakker AHF, 2001. Apolipoprotein E protects against neuropathology induced by a high-fat diet and maintains the integrity of the blood-brain barrier during aging. Laboratory Investigation 81:953–60 10.1038/labinvest.3780307 11454984

[35] Ngai YF, Quong WL, Glier MB, Glavas MM, Babich SL, 2010. Ldlr−/ − mice display decreased susceptibility to Western-type diet-induced obesity due to increased thermogenesis. Endocrinology 151:5226–36 10.1210/en.2010-0496 20881250

[36] Henninger DD, Gerritsen ME, Granger DN. 1997. Low-density lipoprotein receptor knockout mice exhibit exaggerated microvascular responses to inflammatory stimuli. Circulation Research 81:274–81 10.1161/01.res.81.2.274 9242189

[37] Bieghs V, Van Gorp PJ, Wouters K, Hendrikx T, Gijbels MJ, 2012. LDL receptor knock-out mice are a physiological model particularly vulnerable to study the onset of inflammation in non-alcoholic fatty liver disease. PLoS One 7:e30668 10.1371/journal.pone.0030668 PMC3266276 22295101

[38] Chaix A, Lin T, Ramms B, Cutler RG, Le T, 2024. Time-restricted feeding reduces atherosclerosis in LDLR KO mice but not in ApoE knockout mice. Arteriosclerosis, Thrombosis, and Vascular Biology 44:2069–87 10.1161/ATVBAHA.124.320998 PMC11409897 39087348

[39] Paalvast Y, Gerding A, Wang Y, Bloks VW, van Dijk TH, 2017. Male apoE*3-Leiden. CETP mice on high-fat high-cholesterol diet exhibit a biphasic dyslipidemic response, mimicking the changes in plasma lipids observed through life in men. Physiological Reports 5:e13376 10.14814/phy2.13376 PMC5641925 29038350

[40] Groot PH, van Vlijmen BJ, Benson GM, Hofker MH, Schiffelers R, 1996. Quantitative assessment of aortic atherosclerosis in APOE*3 Leiden transgenic mice and its relationship to serum cholesterol exposure. Arteriosclerosis, Thrombosis, and Vascular Biology 16:926–33 10.1161/01.atv.16.8.926 8696955

[41] Lutgens E, Daemen M, Kockx M, Doevendans P, Hofker M, 1999. Atherosclerosis in APOE*3-leiden transgenic mice: from proliferative to atheromatous stage. Circulation 99:276–83 10.1161/01.cir.99.2.276 9892595

[42] Zhang J, Shi Q, Hu Y, Li X. 2022. Silibinin augments the effect of clopidogrel on atherosclerosis in diabetic ApoE deficiency mice. Clinical Hemorheology and Microcirculation 80:353–61 10.3233/CH-211279 34602463

[43] Oppi S, Lüscher TF, Stein S. 2019. Mouse models for atherosclerosis research-which is my line? Frontiers in Cardiovascular Medicine 6:46 10.3389/fcvm.2019.00046 PMC6473202 31032262

[44] Galkina EV, Butcher M, Keller SR, Goff M, Bruce A, 2012. Accelerated atherosclerosis in Apoe^−/−^ mice heterozygous for the insulin receptor and the insulin receptor substrate-1. Arteriosclerosis, Thrombosis, and Vascular Biology 32:247–56 10.1161/ATVBAHA.111.240358 PMC3676303 22199371

[45] Kawashima Y, Chen J, Sun H, Lann D, Hajjar RJ, 2009. Apolipoprotein E deficiency abrogates insulin resistance in a mouse model of type 2 diabetes mellitus. Diabetologia 52:1434–41 10.1007/s00125-009-1378-8 PMC5488278 19436992

[46] Zhang Y, Cheng Z, Hong L, Liu J, Ma X, 2023. Apolipoprotein E (ApoE) orchestrates adipose tissue inflammation and metabolic disorders through NLRP3 inflammasome. Molecular Biomedicine 4:47 10.1186/s43556-023-00158-8 PMC10703753 38062308

[47] Hinder LM, Vincent AM, Hayes JM, McLean LL, Feldman EL. 2013. Apolipoprotein E knockout as the basis for mouse models of dyslipidemia-induced neuropathy. Experimental Neurology 239:102–10 10.1016/j.expneurol.2012.10.002 PMC3534788 23059459

[48] Zabalawi M, Bhat S, Loughlin T, Thomas MJ, Alexander E, 2003. Induction of fatal inflammation in LDL receptor and ApoA-I double-knockout mice fed dietary fat and cholesterol. The American Journal of Pathology 163(3):1201–13 10.1016/S0002-9440(10)63480-3 PMC1868257 12937162

[49] Rensing KL, de Jager SCA, Stroes ES, Vos M, Twickler MTB, 2014. Akt2/LDLr double knockout mice display impaired glucose tolerance and develop more complex atherosclerotic plaques than LDLr knockout mice. Cardiovascular Research 101:277–87 10.1093/cvr/cvt252 24220638

[50] Golforoush P, Yellon DM, Davidson SM. 2020. Mouse models of atherosclerosis and their suitability for the study of myocardial infarction. Basic Research in Cardiology 115:73 10.1007/s00395-020-00829-5 PMC7704510 33258000

[51] Getz GS, Reardon CA. 2006. Diet and murine atherosclerosis. Arteriosclerosis, Thrombosis, and Vascular Biology 26:242–49 10.1161/01.ATV.0000201071.49029.17 16373607

[52] Funes AK, Avena V, Boarelli PV, Monclus MA, Zoppino DF, 2024. Cholesterol dynamics in rabbit liver: high-fat diet, olive oil, and synergistic dietary effects. Biochemical and Biophysical Research Communications 733:150675 10.1016/j.bbrc.2024.150675 39284268

[53] Li Y, Gan M, Tang T, Shao J, Lai T, 2021. Intramuscular adipocyte and fatty acid differences between high-fat and control rabbit groups subject to a restricted diet. Veterinary Medicine and Science 7:2051–60 10.1002/vms3.576 PMC8464271 34273256

[54] Mäkitaipale J, Sievänen H, Sankari S, Laitinen-Vapaavuori O. 2019. Diet is a main source of vitamin D in Finnish pet rabbits (*Oryctolagus cuniculus*). Journal of Animal Physiology and Animal Nutrition 103:1564–70 10.1111/jpn.13120 31152471

[55] Myers DL, Liaw L. 2004. Improved analysis of the vascular response to arterial ligation using a multivariate approach. The American Journal of Pathology 164:43–48 10.1016/S0002-9440(10)63094-5 PMC1602224 14695317

[56] Croatt AJ, Grande JP, Hernandez MC, Ackerman AW, Katusic ZS, 2010. Characterization of a model of an arteriovenous fistula in the rat the effect of L-NAME. The American Journal of Pathology 176:2530–41 10.2353/ajpath.2010.090649 PMC2861117 20363917

[57] Bozzetto M, Rota S, Vigo V, Casucci F, Lomonte C, 2017. Clinical use of computational modeling for surgical planning of arteriovenous fistula for hemodialysis. BMC Medical Informatics and Decision Making 17:26 10.1186/s12911-017-0420-x PMC5348915 28288599

[58] Li Y, Hu K, Li Y, Lu C, Guo Y, 2024. The rodent models of arteriovenous fistula. Frontiers in Cardiovascular Medicine 11:1293568 10.3389/fcvm.2024.1293568 PMC10830807 38304139

[59] Zhao Y, Qu H, Wang Y, Xiao W, Zhang Y, 2020. Small rodent models of atherosclerosis. Biomedicine & Pharmacotherapy 129:110426 10.1016/j.biopha.2020.110426 32574973

[60] Wu H, Gao T, Cao Y, Diao J, Chang F, 2019. Protective and therapeutic effects of *Trianthema portulacastrum* against atherosclerosis in male albino rats *via* G-protein-coupled receptor 124. AMB Express 9:178 10.1186/s13568-019-0901-7 PMC6823334 31673813

[61] Lee JG, Ha CH, Yoon B, Cheong SA, Kim G, 2019. Knockout rat models mimicking human atherosclerosis created by Cpf1-mediated gene targeting. Scientific Reports 9:2628 10.1038/s41598-019-38732-2 PMC6385241 30796231

[62] Spagnoli LG, Orlandi A, Mauriello A, Santeusanio G, de Angelis C, 1991. Aging and atherosclerosis in the rabbit. 1. Distribution, prevalence and morphology of atherosclerotic lesions. Atherosclerosis 89:11–24 10.1016/0021-9150(91)90003-l 1772469

[63] Phinikaridou A, Hallock KJ, Qiao Y, Hamilton JA. 2009. A robust rabbit model of human atherosclerosis and atherothrombosis. Journal of Lipid Research 50:787–97 10.1194/jlr.M800460-JLR200 PMC2666165 19141434

[64] Wu Y, Li F, Wang Y, Hu T, Gao L. 2022. Lipid-lowering treatment in a rabbit model of atherosclerosis: a vessel wall magnetic resonance imaging study. Annals of Translational Medicine 10:569 10.21037/atm-22-1263 PMC9201133 35722437

[65] Fan J, Kitajima S, Watanabe T, Xu J, Zhang J, 2015. Rabbit models for the study of human atherosclerosis: From pathophysiological mechanisms to translational medicine. Pharmacology & Therapeutics 146:104–19 10.1016/j.pharmthera.2014.09.009 PMC4304984 25277507

[66] Hamamdzic D, Wilensky RL. 2013. Porcine models of accelerated coronary atherosclerosis: role of diabetes mellitus and hypercholesterolemia. Journal of Diabetes Research 2013:761415 10.1155/2013/761415 PMC3697774 23844374

[67] Kim M, Kim HB, Park DS, Cho KH, Hyun DY, 2021. A model of atherosclerosis using nicotine with balloon overdilation in a porcine. Scientific Reports 11:13695 10.1038/s41598-021-93229-1 PMC8249376 34211091

[68] Hoogendoorn A, den Hoedt S, Hartman EMJ, Krabbendam-Peters I, Te Lintel Hekkert M, 2019. Variation in coronary atherosclerosis severity related to a distinct LDL (low-density lipoprotein) profile: findings from a familial hypercholesterolemia pig model. Arteriosclerosis, Thrombosis, and Vascular Biology 39:2338–52 10.1161/ATVBAHA.119.313246 PMC6818985 31554418

[69] Getz GS, Reardon CA. 2012. Animal models of atherosclerosis. Arteriosclerosis, Thrombosis, and Vascular Biology 32(5):1104–15 10.1161/ATVBAHA.111.237693 PMC3331926 22383700

[70] Cox LA, Olivier M, Spradling-Reeves K, Karere GM, Comuzzie AG, 2017. Nonhuman Primates and translational research-cardiovascular disease. ILAR Journal 58:235–50 10.1093/ilar/ilx025 PMC6057500 28985395

[71] Angom RS, Nakka NMR. 2024. Zebrafish as a model for cardiovascular and metabolic disease: the future of precision medicine. Biomedicines 12:693 10.3390/biomedicines12030693 PMC10968160 38540306

[72] Tang D, Geng F, Yu C, Zhang R. 2021. Recent application of zebrafish models in atherosclerosis research. Frontiers in Cell and Developmental Biology 9:643697 10.3389/fcell.2021.643697 PMC7947229 33718384

[73] Ouimet M, Franklin V, Mak E, Liao X, Tabas I, 2011. Autophagy regulates cholesterol efflux from macrophage foam cells *via* lysosomal acid lipase. Cell Metabolism 13:655–67 10.1016/j.cmet.2011.03.023 PMC3257518 21641547

[74] Gui Y, Zheng H, Cao RY. 2022. Foam cells in atherosclerosis: novel insights into its origins, consequences, and molecular mechanisms. Frontiers in Cardiovascular Medicine 9:845942 10.3389/fcvm.2022.845942 PMC9043520 35498045

[75] Onodera A, Shimomura T, Ochi H, Sunada R, Fukutomi E, 2023. The cellular accumulation of vehicle exhaust particulates changes the acidic pH environment of lysosomes in BEAS-2B airway epithelial cells. Journal of Xenobiotics 13:653–61 10.3390/jox13040042 PMC10660702 37987443

[76] Xing Y, Lin X. 2024. Challenges and advances in the management of inflammation in atherosclerosis. Journal of Advanced Research10.1016/j.jare.2024.06.016 PMC12126742 38909884

[77] Ait-Oufella H, Libby P. 2024. Inflammation and atherosclerosis: prospects for clinical trials. Arteriosclerosis, Thrombosis, and Vascular Biology 44:1899–905 10.1161/ATVBAHA.124.320155 PMC11343092 39167675

[78] Yousaf H, Khan MIU, Ali I, Munir MU, Lee KY. 2023. Emerging role of macrophages in non-infectious diseases: an update. Biomedicine & Pharmacotherapy 161:114426 10.1016/j.biopha.2023.114426 36822022

[79] Pepin ME, Gupta RM. 2024. The role of endothelial cells in atherosclerosis: insights from genetic association studies. The American Journal of Pathology 194:499–509 10.1016/j.ajpath.2023.09.012 PMC10988759 37827214

[80] Xu J, Kitada M, Ogura Y, Koya D. 2021. Relationship between autophagy and metabolic syndrome characteristics in the pathogenesis of atherosclerosis. Frontiers in Cell and Developmental Biology 9:641852 10.3389/fcell.2021.641852 PMC8083902 33937238

[81] Pan W, Zhang J, Zhang L, Zhang Y, Song Y, 2024. Comprehensive view of macrophage autophagy and its application in cardiovascular diseases. Cell Proliferation 57:e13525 10.1111/cpr.13525 PMC10771119 37434325

[82] Das R, Ganapathy S, Mahabeleshwar GH, Drumm C, Febbraio M, 2013. Macrophage gene expression and foam cell formation are regulated by plasminogen. Circulation 127:1209–18 10.1161/CIRCULATIONAHA.112.001214 PMC3638243 23401155

[83] Sijbesma JWA, van Waarde A, Kristensen S, Kion I, Tietge UJF, 2023. Characterization of a novel model for atherosclerosis imaging: the apolipoprotein E-deficient rat. EJNMMI Research 13:106 10.1186/s13550-023-01055-5 PMC10713960 38079017

[84] Zhang H, Wu LM, Wu J. 2011. Cross-talk between apolipoprotein E and cytokines. Mediators of Inflammation 2011:949072 10.1155/2011/949072 PMC3136159 21772670

[85] Cai Y, Wen J, Ma S, Mai Z, Zhan Q, 2021. Huang-Lian-Jie-du decoction attenuates atherosclerosis and increases plaque stability in high-fat diet-induced ApoE^−/−^ mice by inhibiting M1 macrophage polarization and promoting M2 macrophage polarization. Frontiers in Physiology 12:666449 10.3389/fphys.2021.666449 PMC8445160 34539422

[86] He L, Chen Q, Wang L, Pu Y, Huang J, 2024. Activation of Nrf2 inhibits atherosclerosis in ApoE^−/−^ mice through suppressing endothelial cell inflammation and lipid peroxidation. Redox Biology 74:103229 10.1016/j.redox.2024.103229 PMC11247299 38870781

[87] Mangarova DB, Reimann C, Kaufmann JO, Möckel J, Kader A, 2024. Elastin-specific MR probe for visualization and evaluation of an interleukin-1*β* targeted therapy for atherosclerosis. Scientific Reports 14:20648 10.1038/s41598-024-71716-5 PMC11375012 39232217

[88] Grebe A, Hoss F, Latz E. 2018. NLRP3 inflammasome and the IL-1 pathway in atherosclerosis. Circulation Research 122:1722–40 10.1161/CIRCRESAHA.118.311362 29880500

[89] Go GW, Mani A. 2012. Low-density lipoprotein receptor (LDLR) family orchestrates cholesterol homeostasis. The Yale Journal of Biology and Medicine 85:19–28 PMC3313535 22461740

[90] Rodger EJ, Porteous CM, Jones GT, Legge M, Kleffmann T, 2018. Proteomic analysis of liver from human lipoprotein(a) transgenic mice shows an oxidative stress and lipid export response. BioMed Research International 2018:4963942 10.1155/2018/4963942 PMC6286786 30596094

[91] Wu Y, Song M, Chen W, Xu K, Wu M, 2023. Liraglutide ameliorates oxidized LDL-induced endothelial dysfunction by GLP-1R-dependent downregulation of LOX-1-mediated oxidative stress and inflammation. Redox Report 28:2218684 10.1080/13510002.2023.2218684 PMC10249460 37278349

[92] Wang X, Zhu L, Liu J, Ma Y, Qiu C, 2024. Palmitic acid in type 2 diabetes mellitus promotes atherosclerotic plaque vulnerability via macrophage Dll4 signaling. Nature Communications 15:1281 10.1038/s41467-024-45582-8 PMC10861578 38346959

[93] Bao X, Liang Y, Chang H, Cai T, Feng B, 2024. Targeting proprotein convertase subtilisin/kexin type 9 (PCSK9): from bench to bedside. Signal Transduction and Targeted Therapy 9:13 10.1038/s41392-023-01690-3 PMC10772138 38185721

[94] Shin D, Kim S, Lee H, Lee HC, Lee J, 2024. PCSK9 stimulates Syk, PKCδ, and NF-κB, leading to atherosclerosis progression independently of LDL receptor. Nature Communications 15:2789 10.1038/s41467-024-46336-2 PMC10981688 38555386

[95] Al-Mashhadi RH, Sørensen CB, Kragh PM, Christoffersen C, Mortensen MB, 2013. Familial hypercholesterolemia and atherosclerosis in cloned minipigs created by DNA transposition of a human *PCSK9* gain-of-function mutant. Science Translational Medicine 5:166ra1 10.1126/scitranslmed.3004853 23283366

[96] Yuan F, Guo L, Park KH, Woollard JR, Taek-Geun K, 2018. Ossabaw pigs with a PCSK9 gain-of-function mutation develop accelerated coronary atherosclerotic lesions: a novel model for preclinical studies. Journal of the American Heart Association 7:e006207 10.1161/JAHA.117.006207 PMC5907533 29572319

[97] Agarwala A, Billheimer J, Rader DJ. 2013. Mighty minipig in fight against cardiovascular disease. Science Translational Medicine 5:166fs1 10.1126/scitranslmed.3005369 23283365

[98] Shim J, Al-Mashhadi R, Sørensen C, Bentzon J. 2016. Large animal models of atherosclerosis–new tools for persistent problems in cardiovascular medicine. The Journal of Pathology 238:257–66 10.1002/path.4646 26414760

[99] Sergin I, Evans TD, Zhang X, Bhattacharya S, Stokes CJ, 2017. Exploiting macrophage autophagy-lysosomal biogenesis as a therapy for atherosclerosis. Nature Communications 8:15750 10.1038/ncomms15750 PMC5467270 28589926

